# The relationship between fibrinogen-to-albumin ratio and peripheral arterial disease: A cross-sectional study

**DOI:** 10.1097/MD.0000000000050650

**Published:** 2026-09-11

**Authors:** Wei Zhu, Siliang Zhang, Ying Xiang, Ankang Lv, Xingsheng Li, Die Pu

**Affiliations:** a Department of Respiratory and Endocrinology, Chongqing Medical and Pharmaceutical College, Chongqing, China; b Department of Geriatrics, The Second Affiliated Hospital of Chongqing Medical University, Chongqing, China.

**Keywords:** cross-sectional study, fibrinogen-to-albumin ratio, NHANES, peripheral arterial disease

## Abstract

Early detection of peripheral arterial disease (PAD) is challenging due to its frequently asymptomatic nature, underscoring the need for accessible biomarkers to enhance risk stratification. This study aimed to investigate the relationship between the fibrinogen-to-albumin ratio (FAR), a marker indicative of both inflammatory and anti-inflammatory states, and the prevalence of PAD. Additionally, we assessed its diagnostic performance in comparison with established inflammatory indices. This cross-sectional analysis included 4234 participants from the National Health and Nutrition Examination Survey conducted between 1999 and 2002. PAD was defined as an ankle-brachial index of <0.9. We examined the relationship between FAR and PAD using multiple logistic regression, restricted cubic splines, and subgroup analyses. The predictive performance of FAR was evaluated using the area under the receiver operating characteristic curve (AUC) and compared with that of other inflammatory indices. After adjusting for covariates, a significant positive correlation was found between FAR and PAD prevalence (odds ratio, 1.01; 95% confidence interval, 1.00–1.02; *P* = .003). A clear dose-response relationship was observed, with a strong trend (*P* for trend < .05) across the FAR quartiles. Restricted cubic spline analysis confirmed a linear association between FAR and the risk of PAD (*P* for overall < .001; *P* for nonlinear = .208). The association remained robust across various subgroups, particularly among former smokers, individuals without renal impairment, and those in partnered relationships. Receiver operating characteristic analysis revealed that FAR achieved an AUC of 0.695 (95% confidence interval, 0.663–0.726), with an optimal cutoff of 8.818% (sensitivity, 67.7%; specificity, 64.2%), significantly surpassing the AUCs of the neutrophil-to-lymphocyte ratio (0.561; *P* < .001), C-reactive protein-to-albumin ratio (0.558; *P* < .001), platelet-to-lymphocyte ratio (0.521; *P* < .001), and systemic immune-inflammation index (0.534; *P* < .001). FAR is independently associated with PAD and demonstrates superior discriminatory performance over conventional inflammatory markers. These findings suggest that FAR may serve as a cost-effective screening tool for identifying high-risk individuals, enabling timely vascular assessment in primary care settings.

## 1. Introduction

Peripheral arterial disease (PAD) is a clinical manifestation of systemic atherosclerosis characterized by progressive narrowing and obstruction of the arteries in the lower extremities.^[1]^ While intermittent claudication is considered the classic symptom of PAD, a significant number of affected individuals remain asymptomatic or present with nonspecific symptoms.^[1]^ This often results in delayed recognition and missed opportunities for early intervention. As PAD progresses, some patients may develop chronic limb-threatening ischemia, a severe condition associated with high rates of limb amputation and disability.^[2]^ Moreover, PAD independently increases the risk of major adverse cardiovascular events such as myocardial infarction and stroke.^[3]^ Worldwide, PAD affects an estimated 5.6% to 7.0% of adults aged ≥25 years, with the prevalence sharply rising to 15% to 20% among individuals aged ≥70 years.^[4,5]^ This significantly contributes to morbidity, mortality, and healthcare burden.^[6]^ Although conventional risk factors such as age, smoking, dyslipidemia, and hypertension are well-established,^[2]^ they often lack the discriminatory precision necessary for effective early diagnosis. This highlights the pressing demand for accessible and reliable biomarkers for improving early PAD detection and risk stratification.

Chronic low-grade inflammation plays a crucial role in the initiation and progression of atherosclerosis, which is the underlying pathophysiological process of PAD. Inflammatory pathways contribute to endothelial dysfunction, lipid deposition, plaque development, and vascular remodeling.^[7]^ Several hematologic indices related to inflammation, including the neutrophil-to-lymphocyte ratio (NLR), platelet-to-lymphocyte ratio (PLR), systemic immune-inflammation index (SII), and C-reactive protein-to-albumin ratio (CAR), have been associated with PAD severity and adverse outcomes.^[8–11]^ However, their utility in the early detection of PAD in clinical practice remains relatively limited, underscoring the need for more robust inflammatory markers that can integrate multiple pathogenic pathways to enhance early diagnostic capabilities.

Recently, the fibrinogen-to-albumin ratio (FAR) has emerged as a promising composite biomarker of cardiovascular and cerebrovascular diseases. Fibrinogen, a key acute-phase reactant, reflects systemic inflammation and pro-thrombotic activity,^[12]^ whereas albumin possesses anti-inflammatory and antioxidant properties, inhibiting platelet aggregation.^[13,14]^ Additionally, elevated fibrinogen levels have been linked to insulin resistance,^[15]^ which may accelerate the progression of atherosclerosis. Previous studies have established associations between FAR and the severity of coronary artery disease,^[16]^ as well as functional outcomes and recurrence risk following acute ischemic stroke.^[17]^ However, the relationship between FAR and PAD, particularly regarding its potential role in early detection, has not been comprehensively examined.

Consequently, this study aimed to investigate the association between FAR and the prevalence of PAD among US adults aged 40 years and older, utilizing data from the National Health and Nutrition Examination Survey (NHANES) for 1999 to 2002. By assessing the diagnostic relevance of FAR in a large, nationally representative population, this study sought to provide new evidence to inform early detection strategies and improve risk assessment for PAD in clinical practice.

## 2. Methods

### 2.1. Data source and study population

**Figure 1.**
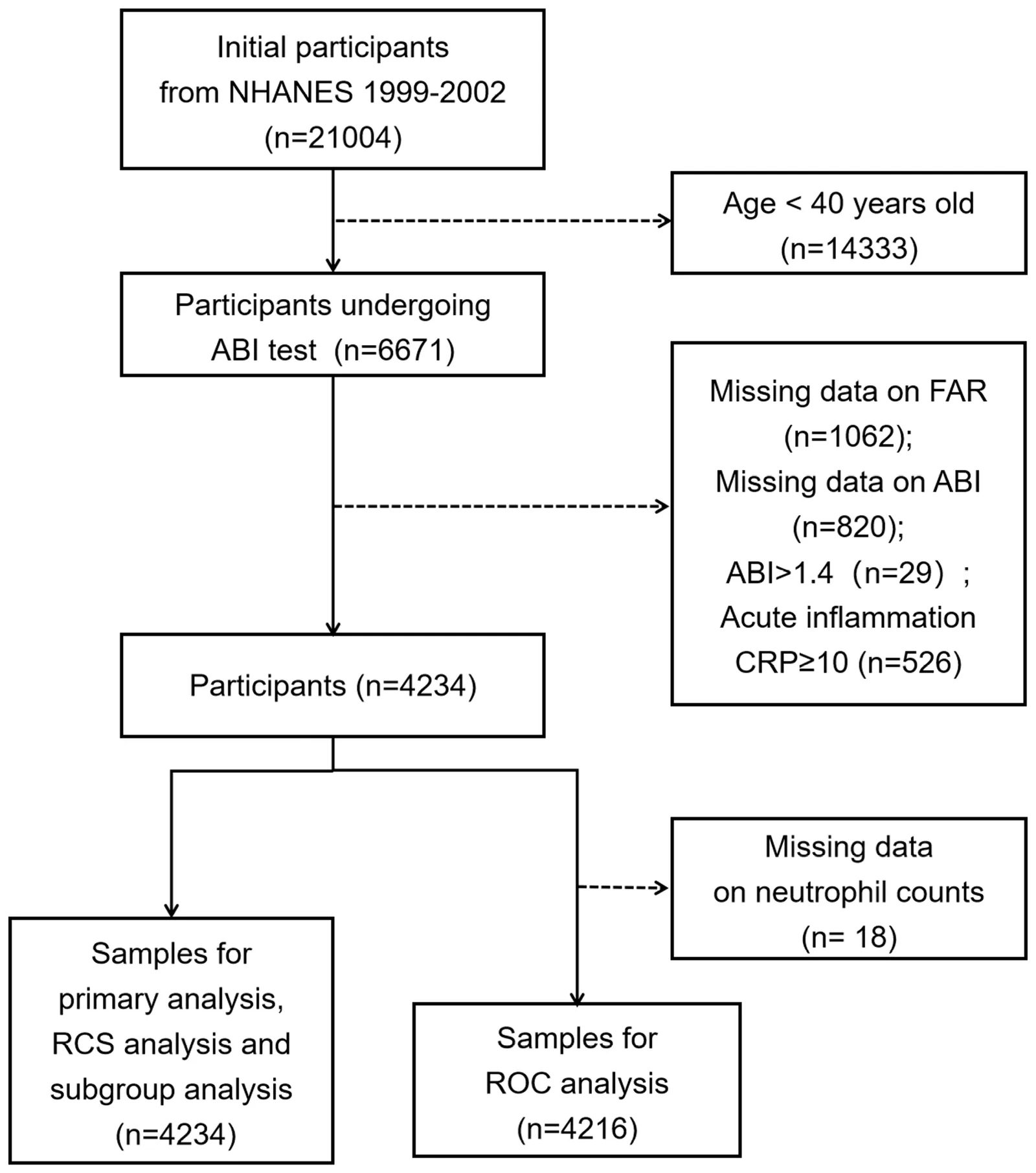
Flowchart of participant selection. ABI = ankle-brachial index, CRP = C-reactive protein, FAR = fibrinogen-to-albumin ratio, NHANES = National Health and Nutrition Examination Survey, RCS = restricted cubic spline, ROC = receiver operating characteristic.

The NHANES is a nationally representative survey conducted by the National Center for Health Statistics (NCHS) in the United States (http://www.cdc.gov/nchs/nhanes.htm). The analysis utilized NHANES data from 1999 to 2002, during which time fibrinogen data were available. The study population comprised 6617 participants aged 40 years or older. We applied the following exclusion criteria sequentially: missing data for FAR (n = 1062), missing data for the ankle-brachial index (ABI; n = 820), ABI >1.4, indicating noncompressible arteries (n = 29), and C-reactive protein (CRP) concentration ≥10 mg/L, indicating acute inflammation (n = 526).^[18]^ After applying these exclusion criteria, 4234 participants were included in the primary analysis to investigate the association between FAR and PAD. For the comparative receiver operating characteristic (ROC) analysis, an additional 18 participants with missing neutrophil counts were excluded, resulting in a final sample of 4216 participants with complete data for FAR, NLR, CAR, PLR, and SII. A flowchart detailing the participant selection is shown in Figure 1.

### 2.2. Exposure

The primary exposure, FAR (%), was calculated using the following formula: (plasma fibrinogen level [g/L]/serum albumin level [g/L]) × 100. We also calculated 4 other inflammatory biomarkers for comparison: NLR (neutrophil count [10^9^/L]/lymphocyte count [10^9^/L]), CAR (CRP [mg/L]/albumin [g/L]) × 100, PLR (platelet count [10^9^/L]/lymphocyte count [10^9^/L]), and SII (platelet count [10^9^/L] × neutrophil count [10^9^/L]/lymphocyte count [10^9^/L]).

### 2.3. Outcomes

PAD was assessed using ABI measurements. The protocol was as follows: systolic pressure was measured in the right brachial artery (or the left arm if contraindications existed) and both ankles (posterior tibial arteries). The ABI for each leg was calculated by dividing the higher ankle systolic pressure by the brachial systolic pressure. A lower ABI value between the 2 legs was used in the analysis. PAD was defined as an ABI of <0.9. As previously stated, participants with an ABI >1.4 in either leg were excluded from all analyses.

### 2.4. Covariates

Potential covariates linked to PAD prevalence were identified based on the weighted univariable logistic regression analysis (shown in Table S1, Supplemental Digital Content 1). These included age (years), sex (male/female), race/ethnicity (categorized as non-Hispanic White, non-Hispanic Black, Mexican American, and Others), educational level (less than high school, high school graduate or above), poverty-income ratio (PIR, continuous), and marital status (partnered or unpartnered). Body mass index (BMI) was calculated as weight in kilograms divided by height in meters squared (kg/m^2^). Smoking status was classified as never, former, or current. Alcohol intake was dichotomized as either drinker or nondrinker. Physical activity was defined as participation in at least 30 minutes of moderate or vigorous activity or strength training 30 or more days preceding the survey. Hypertension was defined as systolic blood pressure ≥140 mm Hg, diastolic blood pressure ≥90 mm Hg, or self-reported physician diagnosis. Hyperlipidemia was defined as 1 or more of the following: total cholesterol ≥5.2 mmol/L, LDL cholesterol ≥3.4 mmol/L, triglycerides ≥1.7 mmol/L, or HDL cholesterol <1.0 mmol/L for males or <1.3 mmol/L for females.^[19,20]^ Renal failure was defined as an estimated glomerular filtration rate <60 mL/min/1.73 m^2^ or a self-reported diagnosis of chronic kidney disease. Cardiovascular disease (CVD) was defined as a self-reported history of coronary heart disease, myocardial infarction, angina, or stroke. Diabetes mellitus was defined as meeting at least one of the following criteria: hemoglobin A1c ≥6.5%, fasting plasma glucose ≥7.0 mmol/L, random plasma glucose ≥11.1 mmol/L, 2-hour oral glucose tolerance test plasma glucose ≥11.1 mmol/L, or current use of insulin or hypoglycemic medications.

### 2.5. Statistical analysis

All statistical analyses were performed using R software (version 4.4.1; R Foundation for Statistical Computing). To account for the complex, multistage probability sampling design of the NHANES, appropriate examination sample weights were applied as per the NCHS guidelines. Continuous variables were presented as weighted mean ± standard error, while categorical variables were presented as unweighted counts (weighted percentages). A two-sided *P* value < .05 was considered statistically significant for all analyses.

#### 2.5.1. Primary analysis

The association between FAR and PAD was assessed using weighted multivariable logistic regression. The results are expressed as odds ratios (ORs) with 95% confidence intervals (CIs). FAR was analyzed as both a continuous variable (per unit increase) and quartiles (Q1–Q4). The quartile cutoff points were determined from the weighted sample distribution as follows: Q1: <6.23; Q2: 6.23 to <7.41; Q3: 7.41 to <8.90; Q4: ≥8.90. A test for trends across quartiles was performed by assigning the median value to each quartile and treating it as a continuous variable in the regression model. Three sequential models were constructed: Model 1 was not adjusted; Model 2 was adjusted for age, sex, race, education, PIR, and marital status; and Model 3 was the fully adjusted model, which included all variables in Model 2 plus BMI, smoking status, alcohol intake, hypertension, CVD, renal failure, hyperlipidemia, and diabetes.

#### 2.5.2. Nonlinearity assessment

The potential nonlinear relationship between continuous FAR and the log-odds of PAD was explored using restricted cubic splines (RCSs) with 3 knots located at the 10th, 50th, and 90th percentiles of the FAR distribution. Nonlinearity was tested using the likelihood ratio test, comparing the model with only the linear term against the model with both the linear and spline terms.

#### 2.5.3. Subgroup analysis and interaction test

To evaluate the robustness of the primary association, we conducted prespecified subgroup analyses. The association between FAR (as a continuous variable) and PAD was examined within strata defined by age (<60 vs ≥60 years), sex, race, PIR (<1.3 vs ≥1.3), education, marital status, smoking status, alcohol intake, BMI (<25, 25–30, ≥30 kg/m^2^), and presence or absence of hypertension, renal failure, CVD, hyperlipidemia, and diabetes. The heterogeneity of the ORs across subgroups was tested by introducing an interaction term between FAR and the subgroup variable into the fully adjusted logistic regression model (Model 3), and its significance was assessed using the Wald test.

#### 2.5.4. Predictive performance comparison

The discriminatory power of FAR for identifying PAD was evaluated and compared with that of NLR, CAR, PLR, and SII using the ROC curve. The area under the ROC curve (AUC) was calculated for each biomarker. Pairwise comparisons of the AUCs were performed using DeLong’s test to determine whether FAR had a statistically superior predictive ability compared with the other indices.

### 2.6. Ethics statement

The NHANES survey protocol was reviewed and approved by the NCHS Ethics Review Board. All participants provided written informed consent.

## 3. Results

### 3.1. Baseline characteristics of the study population

The baseline characteristics of the 4234 study participants, stratified according to PAD status, are summarized in Table 1. Participants with PAD (n = 277) were significantly older, had a lower socioeconomic status (as measured by the PIR), and demonstrated a higher prevalence of comorbidities such as CVD, hypertension, diabetes, and chronic kidney disease than those without PAD (n = 3957). Moreover, the PAD group included a significantly higher proportion of individuals who were physically inactive, had a history of smoking (either former or current), and were nondrinkers. Notably, levels of inflammatory biomarkers, specifically FAR, NLR, and CAR, were significantly elevated in the PAD group, while PLR did not show a significant difference between the groups. The distribution of FAR quartiles revealed that over half (55.6%) of the patients with PAD were in the highest FAR quartile (Q4), in stark contrast to only 27.6% of those without PAD.

**Table 1:** Baseline characteristics of the study population.

Variables	Overall n = 4234	Non-PAD n = 3957	PAD n = 277	*P* value
Age (yr)	59.7 (13.1)	58.9 (12.8)	71.9 (10.9)	<.001
Sex, n (%)				.575
Male	2247 (53.1)	2105 (53.2)	142 (51.3)	
Female	1987 (46.9)	1852 (46.8)	135 (48.7)	
Educational level, n (%)				<.001
Below high school	1464 (34.6)	1335 (33.7)	129 (46.6)	
High school or above	2770 (65.4)	2622 (66.3)	148 (53.4)	
Race, n (%)				.001
Mexican American	948 (22.4)	902 (22.8)	46 (16.6)	
White	2295 (54.2)	2135 (54.0)	160 (57.8)	
Black	692 (16.3)	631 (15.9)	61 (22.0)	
Other	299 (7.06)	289 (7.30)	10 (3.61)	
Marital status, n (%)				<.001
Yes	2738 (64.7)	2588 (65.4)	150 (54.2)	
No	1496 (35.3)	1369 (34.6)	127 (45.8)	
PIR, n (%)				<.001
<1.3	911 (21.5)	822 (20.8)	89 (32.1)	
≥1.3	3323 (78.5)	3135 (79.2)	188 (67.9)	
Alcohol intake, n (%)				.009
Yes	1114 (26.3)	1060 (26.8)	54 (19.5)	
No	3120 (73.7)	2897 (73.2)	223 (80.5)	
Smoking status, n (%)				<.001
Never smoker	1992 (47.0)	1894 (47.9)	98 (35.4)	
Former smoker	1448 (34.2)	1334 (33.7)	114 (41.2)	
Current smoker	794 (18.8)	729 (18.4)	65 (23.5)	
BMI (kg/m^2^)	28.1 (5.36)	28.1 (5.37)	27.2 (5.04)	.003
Renal failure, n (%)				<.001
Yes	484 (11.4)	393 (9.93)	91 (32.9)	
No	3750 (88.6)	3564 (90.1)	186 (67.1)	
Hypertension, n (%)				<.001
Yes	2189 (51.7)	1976 (49.9)	213 (76.9)	
No	2045 (48.3)	1981 (50.1)	64 (23.1)	
Hyperlipidemia, n (%)				.607
Yes	2729 (64.5)	2546 (64.3)	183 (66.1)	
No	1505 (35.5)	1411 (35.7)	94 (33.9)	
Diabetes, n (%)				<.001
Yes	702 (16.6)	616 (15.6)	86 (31.0)	
No	3532 (83.4)	3341 (84.4)	191 (69.0)	
Physical activity, n (%)				<.001
Yes	2395 (56.6)	2284 (57.7)	111 (40.1)	
No	1839 (43.4)	1673 (42.3)	166 (59.9)	
Age classification, n (%)				<.001
[40, 60)	2106 (49.7)	2072 (52.4)	34 (12.3)	
[60, 65)	1442 (34.1)	1329 (33.6)	113 (40.8)	
≥65	686 (16.2)	556 (14.1)	130 (46.9)	
BMI category, n (%)				.055
<25	1228 (29.4)	1134 (29.0)	94 (35.3)	
[25, 30)	1685 (40.4)	1580 (40.4)	105 (39.5)	
≥30	1260 (30.2)	1193 (30.5)	67 (25.2)	
CVD, n (%)				<.001
Yes	542 (12.8)	464 (11.7)	78 (28.2)	
No	3692 (87.2)	3493 (88.3)	199 (71.8)	
FAR (%)	8.43 (1.86)	8.35 (1.83)	9.61 (1.89)	<.001
FAR quartile, n (%)				<.001
Q1	882 (20.8)	865 (21.9)	17 (6.14)	
Q2	944 (22.3)	912 (23.0)	32 (11.6)	
Q3	1160 (27.4)	1086 (27.4)	74 (26.7)	
Q4	1248 (29.5)	1094 (27.6)	154 (55.6)	
NLR	2.15 (1.07)	2.14 (1.05)	2.41 (1.31)	.001
CAR (%)	6.74 (5.63)	6.63 (5.59)	8.31 (5.91)	<.001
PLR	139 (55.5)	139 (55.1)	138 (60.5)	.703
SII	560 (354)	554 (332)	639 (584)	.019

Data are presented as mean ± standard deviation or n (weighted %). *P* values for continuous variables were derived from weighted linear regression, and those for categorical variables were derived from weighted chi-square tests.

BMI = body mass index, CAR = C-reactive protein-to-albumin ratio, CVD = cardiovascular disease, FAR = fibrinogen-to-albumin ratio, NLR = neutrophil-to-lymphocyte ratio, PAD = peripheral arterial disease, PIR = poverty-income ratio (a PIR <1.3 is classified as below the poverty line), PLR = platelet-to-lymphocyte ratio, SII = systemic immune-inflammation index.

### 3.2. Multivariate analysis

The associations between FAR and the risk of PAD were assessed using multivariable logistic regression, and detailed results for the FAR quartiles are shown in Table 2. When analyzed as a continuous variable (per unit increase), FAR remained a significant independent predictor of PAD across all the progressively adjusted models. In the fully adjusted model (Model 3), each unit increase in FAR was associated with a 1% increase in the odds of PAD (OR, 1.01; 95% CI, 1.00–1.02; *P* = .003). The results of the continuous FAR analysis are presented in Table S2, Supplemental Digital Content 2.

**Table 2:** The association between fibrinogen-to-albumin ratio quartile and peripheral arterial disease risk.

	Q1 (<6.923)	Q2 (6.923–8.000)	Q3 (8.000–9.273)	Q4 (≥9.273)	*P* for trend
n	882	944	1160	1248	
Model 1					
OR (95% CI)	Ref	1.89 (0.90–3.97)	4.25 (1.95–9.25)	9.54 (4.37–20.8)	
*P*	Ref	.089	<.001*	<.001*	<.001*
Model 2					
OR (95% CI)	Ref	1.38 (0.63–3.02)	2.63 (1.15–6.03)	4.30 (1.85–9.96)	
*P*	Ref	.405	.025*	.002*	<.001*
Model 3					
OR (95% CI)	Ref	1.26 (0.52–3.02)	2.33 (0.92–5.90)	3.42 (1.26–9.28)	
*P*	Ref	.561	.069	.022*	.005*

Model 1: crude model.

Model 2: adjusted for age, sex, PIR, ethnicity, education, and marital status.

Model 3: Model 2 plus additional adjustment for smoking status, alcohol intake, BMI, physical activity level, disease history of hypertension, hyperlipidemia, renal failure, and CVD.

BMI = body mass index, CI = confidence interval, CVD = cardiovascular disease, OR = odds ratio.

*P < .05.

When evaluated by quartiles, a significant dose-response relationship emerged, marked by a strong positive trend across increasing quartiles (*P* for trend = .005 in Model 3). In the fully adjusted model, participants in the highest FAR quartile (Q4) exhibited a substantially increased risk of PAD compared with those in the lowest quartile (Q1; OR, 3.42; 95% CI, 1.26–9.28; *P* = .022). Although the risk estimates for the second and third quartiles were elevated, they did not achieve statistical significance in the fully adjusted model.

### 3.3. Dose-response relationship between FAR and PAD

**Figure 2.**
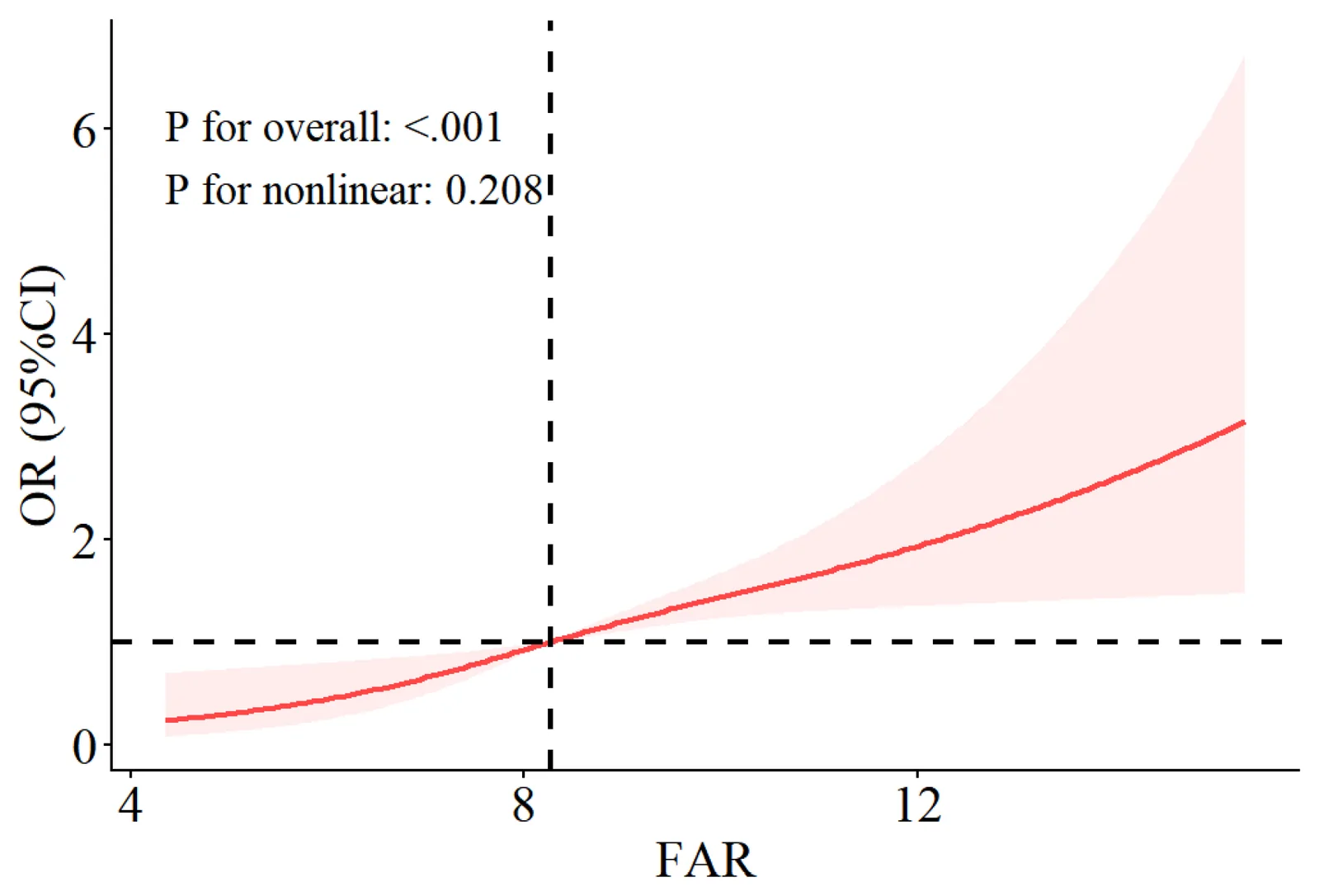
The dose-response relationship between the FAR index and the risk of PAD. Age, sex, race, educational level, marital status, poverty-income ratio, smoking status, alcohol intake, physical activity, body mass index, diabetes, CVD, renal failure, hypertension, and hyperlipidemia were adjusted. CI = confidence interval, CVD = cardiovascular disease, FAR = fibrinogen‐to‐albumin ratio, OR = odds ratio, PAD = peripheral arterial disease.

We utilized RCSs to visualize the potential nonlinear relationship between FAR (as a continuous variable) and the odds of PAD. As illustrated in Figure 2, the analysis indicated a statistically significant linear association between FAR and PAD risk (*P* for overall < .001; *P* for nonlinear = .208).

### 3.4. Subgroup and sensitivity analyses

**Figure 3.**
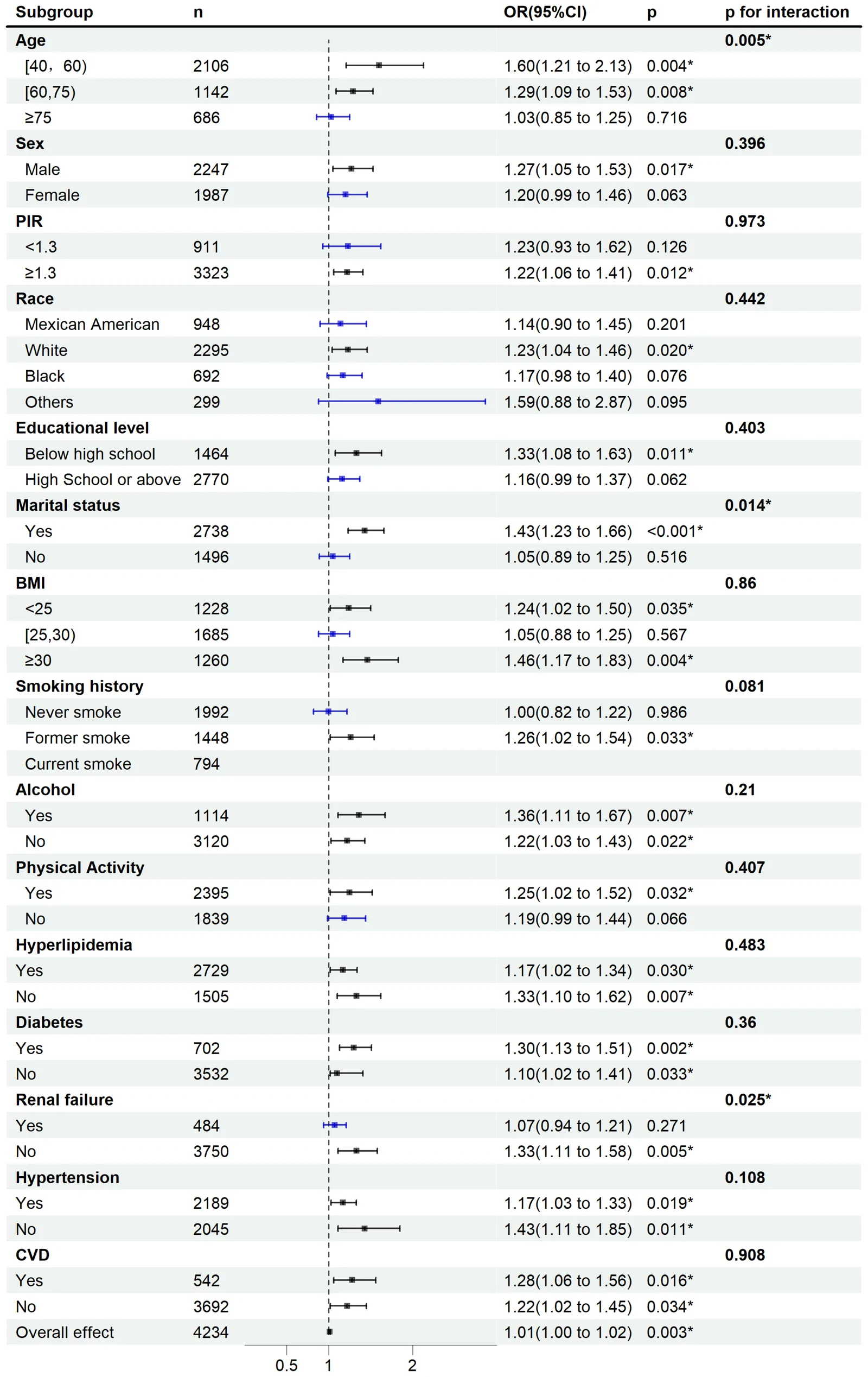
Subgroup analysis and interaction test of FAR and PAD. * indicates *P* < .05. Bold values indicate *P* values for interaction. 95% CI = 95% confidence interval, BMI = body mass index, CVD = cardiovascular disease, FAR = fibrinogen-to-albumin ratio, OR = odds ratio, PAD = peripheral arterial disease, PIR = poverty-income ratio.

The association between FAR and PAD remained robust across a variety of prespecified subgroups, as shown in the forest plot (Fig. 3). Significant positive associations were observed in the majority of the analyzed subgroups, underscoring the stability and widespread nature of this relationship.

Despite this overall consistency, the formal interaction tests revealed that the strength of the association was significantly modified by age (*P* = .005), marital status (*P* = .014), and renal failure status (*P* = .025).

### 3.5. ROC curve comparison of inflammatory biomarkers

**Figure 4.**
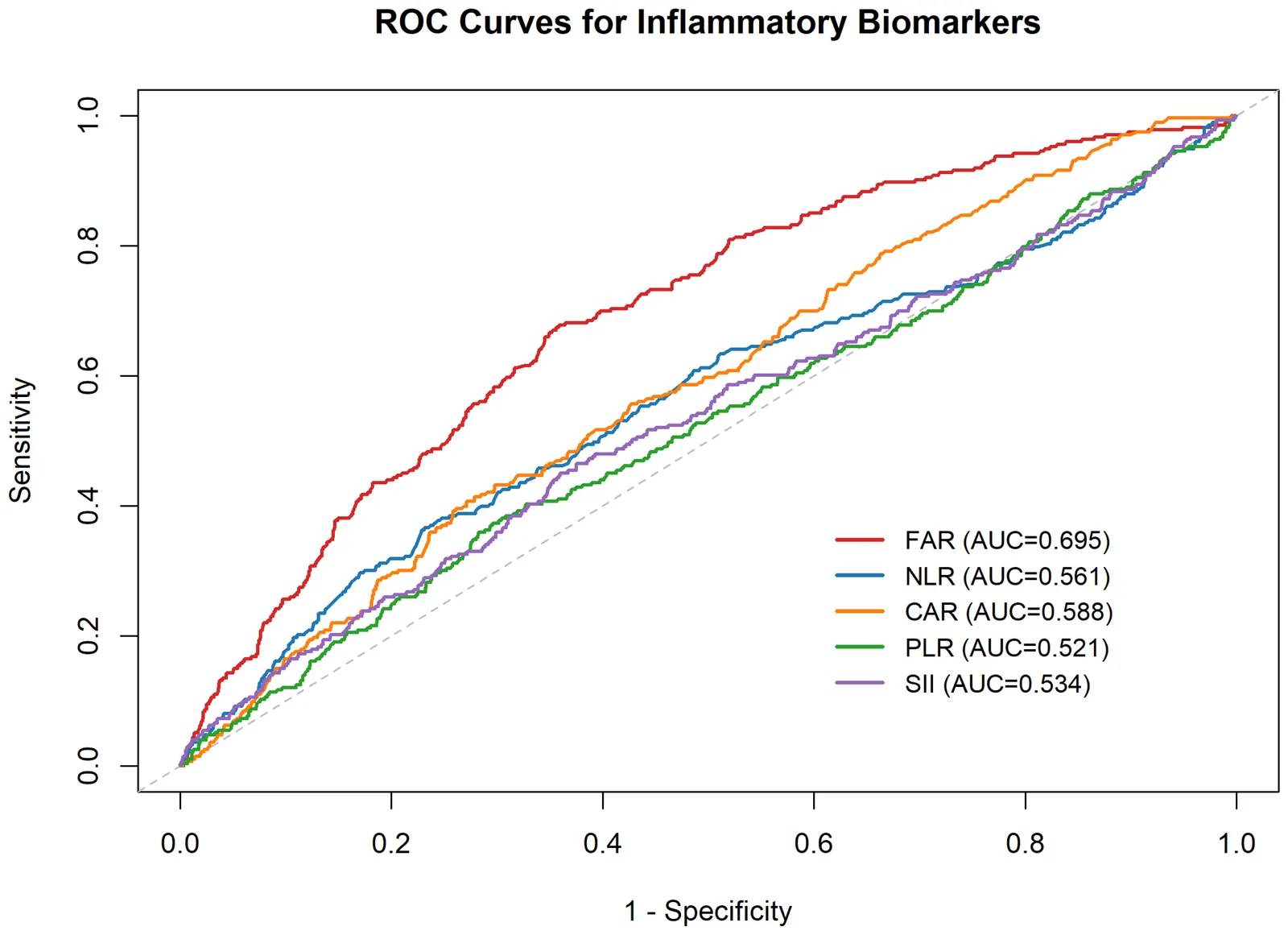
Receiver operating characteristic curve for the diagnostic accuracy of FAR, NLR, CAR, PLR, and SII in PAD. AUC = area under the receiver operating characteristic curve, CAR = C-reactive protein-to-albumin ratio, FAR = fibrinogen‐to‐albumin ratio, NLR = neutrophil-to-lymphocyte ratio, PAD = peripheral arterial disease, PLR = platelet-to-lymphocyte ratio, ROC = receiver operating characteristic, SII = systemic immune-inflammation index.

We evaluated the predictive performance of FAR for PAD in comparison with other inflammatory biomarkers, including NLR, CAR, PLR, and SII, within a cohort of 4216 participants. As illustrated in Figure 4, the AUC for FAR was found to be 0.695 (95% CI, 0.663–0.726), significantly outperforming the AUCs of the other biomarkers: NLR (0.588; 95% CI, 0.554–0.621; *P* < .001), CAR (0.554; 95% CI, 0.493–0.625; *P* < .001), PLR (0.521; 95% CI, 0.484–0.558; *P* < .001), and SII (0.534; 95% CI, 0.497–0.572; *P* < .001). This finding underscores the superior predictive ability of FAR. The optimal cutoff value of FAR for identifying PAD was 8.818%, with a sensitivity of 67.7% and a specificity of 64.2%. The corresponding values for all biomarkers are summarized in Table S3, Supplemental Digital Content 3.

## 4. Discussion

This study investigated the relationship between the FAR and the prevalence of PAD in a nationally representative sample of adults in the US. We found that an elevated FAR was significantly and independently associated with an increased PAD risk. This relationship remained robust across multiple analytical approaches, including multivariable regression, quartile-based trend analysis, and RCS modeling, which collectively demonstrated a clear dose-response pattern and a linear association between FAR and PAD. The stability of this association across extensive subgroup analyses further reinforced its reliability. Notably, FAR also exhibited superior diagnostic performance compared with several widely used inflammatory biomarkers, including NLR, CAR, PLR, and SII, as indicated by ROC curve analysis. Taken together, our findings suggest that FAR is a readily available, cost-effective, and powerful biomarker that may meaningfully enhance early PAD risk stratification in community-based and primary care settings.

PAD represents a chronic atherosclerotic condition driven by progressive vascular inflammation, endothelial dysfunction, and thrombosis. Fibrinogen, a pro-inflammatory and pro-thrombotic plasma protein, contributes to atherosclerotic plaque formation, vascular injury, coagulation, and thrombosis by stimulating pro-inflammatory cytokine release (e.g., interleukin-1 and tumor necrosis factor-alpha), enhancing monocyte/macrophage adhesion, increasing blood viscosity, and facilitating platelet aggregation.^[7,21]^ Previous studies have suggested that higher serum fibrinogen concentration is independently associated with both prevalence and incidence of PAD.^[22–24]^ In contrast, serum albumin exhibits anti-inflammatory, antioxidant, and endothelial-protective functions,^[13,14]^ and low albumin levels have been linked to a higher risk of coronary artery disease and PAD.^[25,26]^ Because FAR integrates these opposing biological influences – elevated fibrinogen-mediated vascular injury and diminished albumin-related vascular protection – it has emerged as a sensitive marker reflecting the cumulative burden of atherogenic and inflammatory stress. Recent studies have shown that higher FAR correlates with the severity of stable coronary artery disease,^[27]^ acute coronary syndrome,^[16]^ and recurrence and poor outcomes after large-artery atherosclerotic stroke.^[17]^ A cross-sectional study with 413 patients with type 2 diabetes mellitus also suggested that FAR was independently and positively correlated with brachial-ankle pulse wave velocity and arterial stiffness.^[28]^ However, evidence for its predictive utility in lower-extremity PAD is limited. A recent study of 280 patients suspected of having PAD who underwent peripheral angiography demonstrated a significant association between FAR and PAD^[29]^; however, population-level data are lacking. Our study expands this evidence by confirming a strong FAR–PAD association in a large, nationally representative cohort, underscoring FAR’s potential value in early PAD identification beyond specialized clinical environments.

We also observed a clear dose-response relationship: PAD prevalence increased significantly across FAR quartiles in all models (*P* for trend < .05), and spline analyses confirmed a linear association (*P* for overall < .001; *P* for nonlinear = .208). The lack of significant nonlinearity may be attributed to the sample size and distribution of FAR values in our cohort, which may have limited the ability to detect subtle threshold effects. Future studies with larger and more diverse populations may reveal threshold or plateau effects that were not detectable in the present analysis. Nevertheless, a FAR value of 8.818% may serve as a useful reference for identifying individuals at elevated risk in primary care settings, while clinical decision-making should incorporate FAR as a continuous variable reflecting its linear dose-response relationship with PAD.

Subgroup analysis not only confirmed the robustness of the primary findings regarding the positive association between FAR and PAD but also identified potential effect modifiers. Smoking, a well-established promoter of chronic vascular injury, inflammation, and atherogenesis,^[30]^ strengthened the association between FAR and PAD, especially among former smokers. This finding suggests that cumulative smoking exposure may amplify the biological consequences of elevated FAR.^[30,31]^ We also found a significant interaction with renal function, wherein the association between FAR and PAD was stronger in participants without renal failure. This may be explained by the fact that in advanced kidney disease, PAD risk is driven by multiple factors, such as uremic toxicity and profound mineral bone disease,^[32,33]^ which may diminish the relative contribution of FAR. Furthermore, a significant interaction was observed with marital status, with a stronger association between FAR and PAD among married and partnered individuals. One possible explanation is that unpartnered individuals often experience less social support, higher levels of psychosocial stress, and unhealthier behavior,^[34]^ leading to elevated systemic inflammation and cardiovascular risk,^[35]^ thereby reducing the discriminatory ability of FAR. Thus, our findings suggest that FAR may hold greater clinical predictive value for PAD specifically in individuals with a history of smoking, preserved renal function, and those who are married. Future large-scale studies are needed to validate the application of FAR in different populations and to explore whether subgroup-specific FAR cutoff values could optimize risk stratification.

Our study adds to the expanding literature linking systemic inflammation with PAD progression and prognosis.^[8,10,11,35–38]^ Consistent with previous research, various inflammatory hematological indices, such as NLR, PLR, and SII, have demonstrated significant associations with PAD severity and adverse clinical outcomes. For instance, a meta-analysis confirmed that elevated NLR predicts poor 1-year outcomes in PAD, including major adverse cardiovascular and limb events.^[8]^ Similarly, other studies have linked NLR and PLR to angiographic severity, gangrene, and amputation risk in patients with PAD,^[10,38]^ whereas SII has been associated with PAD in patients with type 2 diabetes.^[11]^ The novel contribution of our analysis lies in the direct comparison of FAR with established markers using the same population. We found that FAR demonstrated superior discriminatory performance, likely because it captured both pro-inflammatory and anti-inflammatory biological pathways. A key practical advantage of FAR is that it is derived from 2 routine, low-cost laboratory parameters, making it an economically feasible and readily accessible tool for widespread clinical implementation. Therefore, patients with a high FAR, particularly those with a smoking history, preserved renal function, or a stable social environment, should be prioritized for more vigilant PAD screening and aggressive management of modifiable cardiovascular risk factors.

This study has several strengths. The utilization of the NHANES database, with its rigorous sampling methodology and standardized data collection procedures, ensures the generalizability of our findings to the broader US adult population. Methodological rigor, including multivariable adjustment, demonstration of a dose-response pattern through both quartile and spline analyses, systematic subgroup exploration, and comparative evaluation of multiple inflammatory biomarkers, provides a robust foundation supporting our conclusions.

However, the limitations of this study must be acknowledged. First, the cross-sectional design prevented us from establishing a temporal or causal relationship between FAR and PAD. Second, despite extensive adjustment, residual confounding cannot be entirely ruled out. Third, PAD diagnosis relied solely on ABI, a noninvasive but non-gold-standard method, potentially introducing misclassification bias, especially in cases of noncompressible arteries. Fourth, our study population was restricted to adults aged ≥40 years, which may limit the generalizability of our findings to other populations. Finally, although FAR showed promising diagnostic performance, optimal cutoff values and longitudinal predictive capacity require validation in prospective cohorts.

## 5. Conclusion

In summary, this study identified FAR as a strong and independent biomarker associated with PAD in adults in the US. FAR outperformed several commonly used inflammatory indices and demonstrated consistent associations across diverse analytical methods. These findings support FAR as a clinically meaningful and readily implementable tool for early PAD risk assessment. Individuals with elevated FAR levels may benefit from targeted vascular evaluation and aggressive prevention strategies. Future longitudinal and interventional studies are warranted to establish FAR-based thresholds and to evaluate their potential role in improving PAD screening and clinical outcomes.

## Acknowledgments

We thank all the participants in this study.

## Author contributions

**Conceptualization:** Xingsheng Li, Die Pu.

**Data curation:** Ying Xiang, Die Pu.

**Formal analysis:** Die Pu.

**Funding acquisition:** Wei Zhu.

**Investigation:** Die Pu.

**Methodology:** Wei Zhu, Siliang Zhang, Ying Xiang.

**Project administration:** Xingsheng Li.

**Software:** Siliang Zhang, Die Pu.

**Supervision:** Wei Zhu, Siliang Zhang.

**Validation:** Siliang Zhang, Ankang Lv.

**Visualization:** Die Pu.

**Writing – original draft:** Die Pu.

**Writing – review & editing:** Wei Zhu, Ankang Lv.






