# Supplementary material for: The relationship between fibrinogen-to-albumin ratio and peripheral arterial disease: A cross-sectional study
Source: Medicine (Baltimore). 2026 Sep 11;105(37):e50650. doi: 10.1097/MD.0000000000050650 (PMC13574487; doi:10.1097/MD.0000000000050650)
Supplement: Supplementary file 1 [file medi-105-e50650-s001.docx]

**Supplementary Table S1. Univariable Logistic Regression Analysis for Factors Associated with PAD**

| Variable | **β** (Coefficient) | SE (Standard Error) | P |
| --- | --- | --- | --- |
| FAR | 0.019 | 0.002 | < .001 *** |
| Gender(Female vs Male) | 0.011 | 0.008 | .147 |
| Race (White vs  Mexican American) | 0.011 | 0.007 | .108 |
| Race (Black vs  Mexican American) | 0.030 | 0.012 | .018 * |
| Race (Other vs  Mexican American) | -0.019 | 0.010 | .063 |
| Education (High School or above vs below) | -0.044 | 0.012 | < .001 *** |
| Age | 0.004 | <0.001 | < .001 *** |
| Marital status (No  vs Yes) | 0.021 | 0.008 | .009 ** |
| PIR (Not poor vs poor) | -0.049 | 0.009 | < .001 *** |
| Diabetes (No vs Yes) | -0.061 | 0.014 | < .001*** |
| Smoke (Former vs no) | 0.027 | 0.007 | < .001 *** |
| Smoke (Current vs no) | 0.029 | 0.007 | < .001 *** |
| Hypertension (No vs Yes) | -0.050 | 0.009 | < .001 *** |
| RF (No vs Yes) | -0.129 | 0.025 | < .001 *** |
| BMI | -0.001 | <0.001 | .141 |
| CVD (No vs Yes) | -0.087 | 0.017 | < .001 *** |
| Hyperlipidemia (No vs Yes) | -0.001 | 0.006 | .867 |
| Physical active (No vs Yes) | 0.042 | 0.010 | < .001 *** |
| Alcohol (No vs Yes) | 0.021 | 0.007 | .005 ** |

PAD = Peripheral Arterial Disease, PIR = poverty-income ratio, a PIR <1.3 is classified as below the poverty Line. BMI = body mass index, CVD = cardiovascular diseases.

Data are presented as regression coefficients (β) with standard errors (SE). * p < 0.05, ** p < 0.01, *** p < 0.001.
