# Supplementary material for: The relationship between fibrinogen-to-albumin ratio and peripheral arterial disease: A cross-sectional study
Source: Medicine (Baltimore). 2026 Sep 11;105(37):e50650. doi: 10.1097/MD.0000000000050650 (PMC13574487; doi:10.1097/MD.0000000000050650)
Supplement: Supplementary file 2 [file medi-105-e50650-s002.docx]

**Supplementary Table S2. Multiple Logistic Regression Analysis for Factors Associated with PAD**

| Variable | β (Coefficient) | SE (Standard Error) | P |
| --- | --- | --- | --- |
| FAR | 0.011 | 0.003 | .003** |
| Gender(Female vs Male) | 0.002 | 0.006 | .616 |
| Race (White vs  Mexican American) | <0.001 | 0.012 | .976 |
| Race (Black vs  Mexican American) | 0.012 | 0.012 | .336 |
| Race (Other vs  Mexican American) | -0.031 | 0.010 | .011 * |
| Education (High School or above vs below) | -0.004 | 0.017 | .835 |
| Age | 0.002 | <0.001 | < .001 *** |
| Marital status (No vs Yes) | -0.001 | 0.007 | .948 |
| PIR (Not poor vs poor) | -0.019 | 0.011 | .134 |
| Diabetes (No vs Yes) | -0.026 | 0.013 | .077 |
| Smoke (Former vs no) | 0.019 | 0.007 | .021* |
| Smoke (Current vs no) | 0.031 | 0.008 | .003 ** |
| Hypertension (No vs Yes) | -0.012 | 0.008 | .139 |
| Alcohol (No vs Yes) | 0.005 | 0.008 | .542 |
| RF (No vs Yes) | -0.05 | 0.021 | .024 * |
| BMI | -0.002 | 0.001 | .009 ** |
| CVD (No vs Yes) | -0.032 | 0.014 | .051 |
| Hyperlipidemia (No vs Yes) | -0.001 | 0.005 | .932 |

PAD = Peripheral Arterial Disease, PIR = poverty-income ratio, a PIR <1.3 is classified as below the poverty Line. BMI = body mass index, CVD = cardiovascular diseases, FAR = fibrinogen to albumin ratio.

Data are presented as regression coefficients (β) with standard errors (SE) from the fully adjusted model. * p < 0.05, ** p < 0.01, *** p < 0.001.
