# Supplementary material for: The relationship between fibrinogen-to-albumin ratio and peripheral arterial disease: A cross-sectional study
Source: Medicine (Baltimore). 2026 Sep 11;105(37):e50650. doi: 10.1097/MD.0000000000050650 (PMC13574487; doi:10.1097/MD.0000000000050650)
Supplement: Supplementary file 3 [file medi-105-e50650-s003.docx]

**Supplementary Table S3. ROC Analysis of Inflammatory Biomarkers for PAD Detection**

| Biomarker | AUC (95% CI) | Optimal Cut-off | Sensitivity (%) | Specificity (%) |
| --- | --- | --- | --- | --- |
| FAR (%) | 0.695 (0.663-0.726) | 8.818 | 67.7 | 64.2 |
| NLR | 0.561 (0.523-0.600) | 2.64 | 36.6 | 76.9 |
| CAR (%) | 0.588 (0.554-0.621) | 8.95 | 40.7 | 72.9 |
| PLR | 0.521 (0.484-0.558) | 108.4 | 70.4 | 37.4 |
| SII | 0.534 (0.497-0.572) | 577.8 | 45.1 | 64.0 |

FAR = fibrinogen‐to‐albumin ratio, NLR = neutrophil-to-lymphocyte ratio, PLR = platelet-to-lymphocyte ratio, SII = systemic immune-inflammation index, CAR = C-reactive protein-to-albumin ratio, AUC = area under the receiver operating characteristic curve.

Data are presented as area under the curve (AUC) with 95% confidence intervals, optimal cutoff values, sensitivity, and specificity. Optimal cutoff values were determined by the Youden index.
